# Prevention and Management of Complications of and Training for Colorectal Endoscopic Submucosal Dissection

**DOI:** 10.1155/2013/287173

**Published:** 2013-06-03

**Authors:** Naohisa Yoshida, Nobuaki Yagi, Yutaka Inada, Munehiro Kugai, Akio Yanagisawa, Yuji Naito

**Affiliations:** ^1^Department of Molecular Gastroenterology and Hepatology, Graduate School of Medical Science, Kyoto Prefectural University of Medicine, 465 Kajii-cho, Kawaramachi-Hirokoji, Kamigyo-ku, Kyoto 602-8566, Japan; ^2^Department of Surgical Pathology, Graduate School of Medical Science, Kyoto Prefectural University of Medicine, 465 Kajii-cho, Kawaramachi-Hirokoji, Kamigyo-ku, Kyoto 602-8566, Japan

## Abstract

Endoscopic submucosal dissection (ESD) is reported to be an efficient treatment with a high rate of *en bloc* resection for large colorectal tumors in Japan and some other Western and Asian countries. ESD is considered less invasive than laparoscopic colectomy. However, ESD carries a higher risk of perforation than endoscopic mucosal resection (EMR). Various devices and training methods for colorectal ESD have been developed to solve the difficulties. In this review, we describe the complications of colorectal ESD and prevention of those complications. On the other hand, colorectal ESD is difficult for less-experienced endoscopists. The unique step-by-step ESD training system is performed in Japan. Additionally, appropriate training, including animal model training, for colorectal ESD should be acquired before working on clinical cases.

## 1. Introduction

Endoscopic submucosal dissection (ESD) is reported to be an efficient treatment with a high rate of *en bloc* resection for large colorectal tumors in Japan and some other Western and Asian countries. The rate of *en bloc* resection for large colorectal tumors by ESD was reported to be 80–98.9% [1–9]. ESD is considered less invasive than laparoscopic colectomy. However, ESD carries a higher risk of perforation than endoscopic mucosal resection (EMR) [7, 8] owing to its associated technical difficulties. First, the colon is winding in nature and has many folds. Moreover, the wall of the colon is thinner than the gastric wall. Various devices and training methods for colorectal ESD have been developed to solve the difficulties. In this review, we describe the complications of colorectal ESD, prevention of those complications, and training for the procedure.

## 2. Safe and Efficient Strategy and Our Therapeutic Results

**Figure 1:**
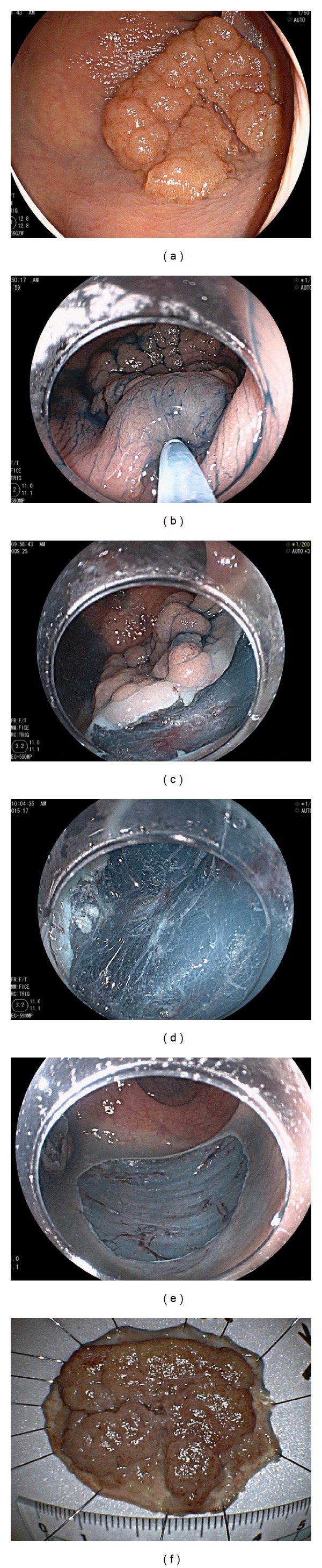
Strategy for endoscopic submucosal dissection. (a) 0-IIa tumor, 30 mm, located in the rectum. First, the tumor and its margin were observed carefully. (b) Injection was performed at the anal (distal) side of the tumor. (c) A partial circumferential mucosal incision was made. (d) Submucosal dissection was performed. (e) The tumor was resected *en bloc*. (f) Resected specimen. Histopathological diagnosis: intramucosal cancer, margin: negative.

The following are the steps in our routine ESD procedure (Figure 1) [3, 7, 8]. First, injection for submucosal elevation is performed with a 25G needle (8B27A; TOP, Tokyo, Japan) after visualization of the border of the tumor, and mucosal incisions are made. A partial circumferential incision is made on the distal side of the tumor. If the tumor is >50 mm in size, the incision is performed at the proximal side of the tumor because in large tumors, it is sometimes difficult to resect the residual mucosa on the proximal side in the presence of a partially resected tumor. Mucosal incisions are performed with the endocut mode (endocut I, effect 2, duration 2, interval 1 in VIO300D; Erbe Elektromedizin Ltd., Tubingen, Germany). Then, simultaneously, an incision into the deep submucosa is made. After the mucosal and submucosal incisions, the submucosa below the tumor is dissected from the distal side of the tumor. Dissection of the submucosa is performed using the endocut mode (endocut I, effect 2, duration 2, interval 1 in VIO300D) or the coagulation mode (swift coagulation, output 40 W, effect 3 in VIO300D). Additional injections are done to achieve submucosal elevation. Half of the dissection is finished, then an oral circumferential mucosal incision is perfomred. And, dissection is continued from the anal side while carefully avoiding perforation and hemorrhage until *en bloc* resection of the tumor is completed.

From 2006 to 2013, we performed ESD in 530 tumors and achieved successful ESD in 517 tumors (97.5%). The average age of patients was 67.5 years (range, 32–92 years) (Table 1). The average tumor size was 31.2 mm (range, 12–130 mm), and the average operation time was 93.4 min (range, 10–420 min). Of the 530 tumors, 364 were located in the colon and 166 were in the rectum. The rate of *en bloc* resection was 91.1%. The histopathological diagnosis was as follows: 236 tumors were adenoma, 218 tumors were intramucosal carcinoma, and 63 tumors were carcinoma with submucosal invasion. Concerning complications, the rate of perforation was 4.1% (i.e., 22 of the 530 tumors). Twenty-one cases were treated only by endoscopic clipping and by withholding the initiation of oral intake, without performing emergency surgery. However, 1 case received emergency surgery. The rate of postoperative hemorrhage was 2.3% (i.e., 12 of the 530 tumors). Cases with postoperative hemorrhage were treated by endoscopic clipping (6 cases) and coagulation with hemostatic forceps (6 cases). Moreover, in these cases, oral intake was withheld and blood transfusion was not given.

## 3. Devices

**Figure 2:**
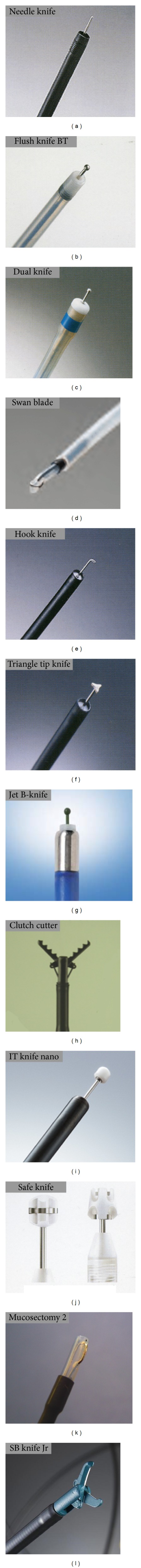
Various knives used for endoscopic submucosal dissection.

A transparent short hood (TOP Co. Ltd., Tokyo, Japan) is fitted at the tip of the endoscope [8]. This helps the easy placement of the endoscope especially during tumor dissection. A special short thin hood (ST hood; Fujifilm Co. Ltd., Tokyo, Japan) is used if the dissection is difficult because of severe fibrosis. A mixture of 0.4% hyaluronic acid solution (Mucoup; Johnson & Johnson K.K., Tokyo, Japan, and Seikagaku Corporation, Tokyo, Japan) is used as the injection liquid to induce greater submucosal elevation and to lengthen the duration of the elevation [8, 10, 11]. Various knives are used in ESD for excising colorectal tumors (Figure 2). Among the obtuse short-tipped types are the Flush knife BT (Fujifilm Medical, Tokyo, Japan), Dual knife (Olympus Optical Co., Tokyo, Japan), and Jet B-knife (Zeon Medical, Tokyo, Japan). The Flush knife BT and Jet B-knife are capable of submucosal injections and omit the need for the endoscopist to switch between the knife and the injection needle [12]. The Dual knife, Jet B-knife, and Flush knife BT all have a ball disk at the tip, enabling the operator to hook the submucosa. The insulated tip (IT) knife nano (Olympus Optical) is being used in certain institutions. The IT knife nano allows rapid dissection. A hook knife (Olympus Optical) is particularly useful when the dissection of the submucosa is difficult owing to poor elevation of the submucosa [6]. The Jet B-knife is the only bipolar knife available, and this knife is believed to cause less burning of the muscularis propria layer compared with other monopolar knives. The clutch cutter (Fujifilm Medical) and SB knife Jr (Sumitomo Bakelite Co., Tokyo, Japan) are grasping-type scissor forceps [13, 14]. At our institution, Flush knife BT is mainly used because it can effectively administer local injections, and the clutch cutter is used secondarily when the risk of perforation is high because of poor submucosal elevation [15]. 

## 4. Complications

### 4.1. Perforation

**Figure 3:**
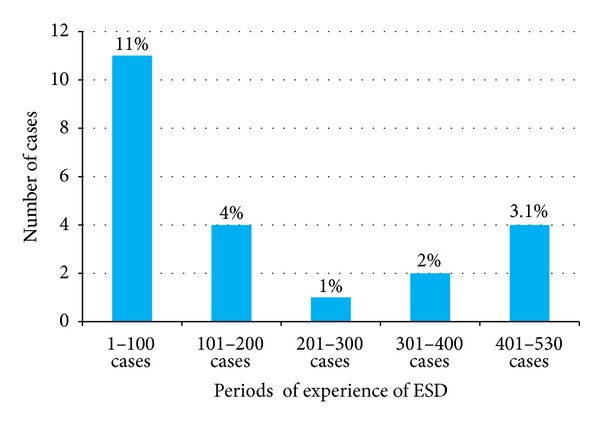
Rate of perioperative perforation in every 100 cases of colorectal endoscopic submucosal dissection (ESD).

**Figure 4:**
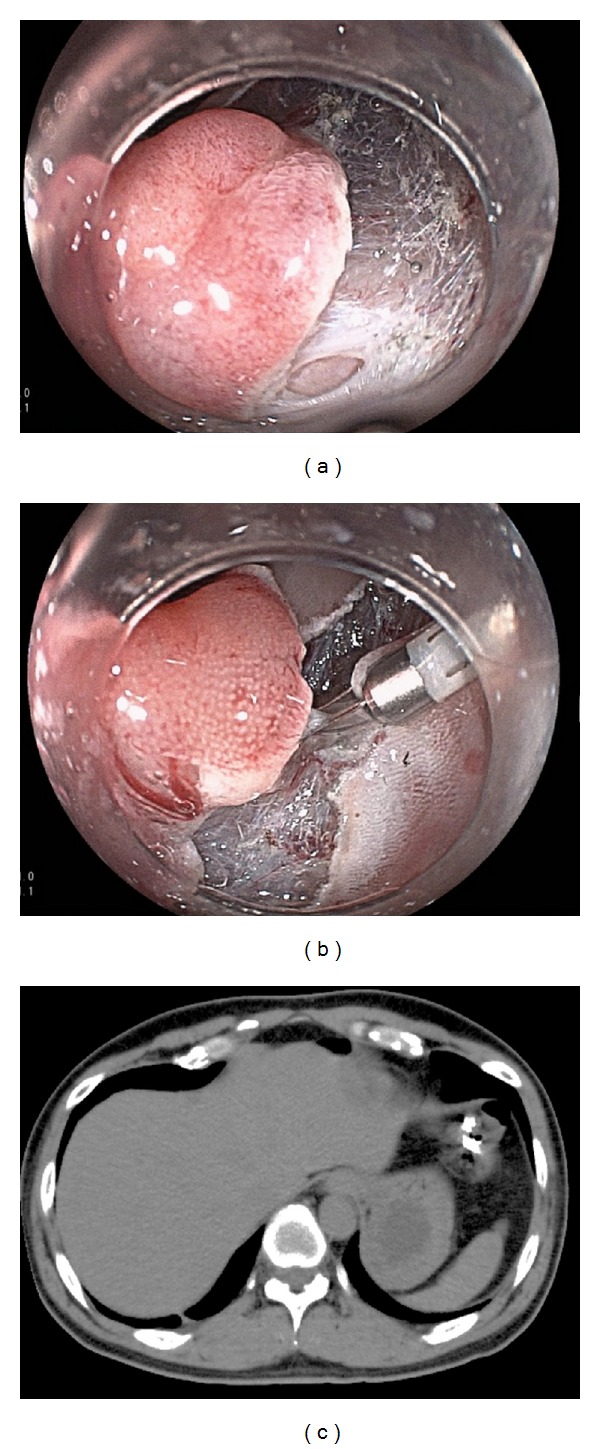
(a) Perforation during dissection. (b) The perforation was closed by endoscopic clipping. (c) Computed tomography revealed the presence of free air outside of the colorectum.

One of the main complications of ESD is perforation, similar to that of EMR. In particular, the rate of perforation is higher for ESD than for EMR (1.5–10.4%) [1–9]. Perforation of the colon can cause fatal peritonitis. Our analysis and other reports have shown that the rate of perforation decreases with increasing experience of the endoscopist [8, 9] (Figure 3). Moreover, Saito et al. [9] reported that the risk of perforation was related to the number of ESD procedures performed; that is, the risk is higher when the endoscopist had performed <100 procedures. Knife coagulation is the most frequent cause of perforation [7]. Among the rare causes of perforation include resection with a snare, use of special hemostat forceps with soft coagulation, and endoscopic clipping onto the coagulated submucosa [7]. Another report revealed that perforation is associated with a large tumor size (>30 mm) and the presence of fibrosis [2]. The paradoxical movement of the endoscope during ESD due to the winding nature of the colorectum causes coagulation in the muscularis propria. A longer operation time increases the amount of air in the abdomen, causing greater paradoxical movement of the endoscope. Concerning knife types, obtuse short-tipped types such as the Dual knife and the Flush knife can cause perforation. In contrast, scissor-type knives such as the clutch cutter and SB knife Jr are less likely to cause perforations because these knives enable grasping the tissue and cutting safely. Previous reports have shown that small perforations can be closed by endoscopic clipping [8, 16]. Thus, most cases of perforation are treated conservatively by endoscopic clipping, without the need for urgent surgical intervention (Figure 4). 

On the other hand, delayed perforation has been reported as a serious complication after ESD [2]. The rate of delayed perforation is reported to be 0.3–0.7% [2, 4, 17]. The reasons for delayed perforation are unknown; however, this type of perforation is reported to be related to excessive coagulation in the muscularis propria. Delayed perforations are reported to be typically large and require treatment by emergency surgery. 

### 4.2. Hemorrhage during ESD and Postoperative Hemorrhage

When a vessel <2 mm in diameter is detected in the submucosa during ESD, it is cut with a knife in the swift coagulation mode (Output 40 W, effect 3 in VIO300D) to prevent hemorrhage. When a vessel >2 mm in diameter is detected, special hemostatic forceps (e.g., Coagrasper; FD-410LR, Olympus Optical) are used in the soft coagulation mode (Output 60 W, effect 5 in VIO300D) to prevent hemorrhage during ESD [18]. These forceps can be rotated, and they are used to gently catch and lift the vessels upward from the muscularis propria. At our institution, hemostatic forceps have been uniquely adopted for use in resecting vessels [18]. In brief, the vessel is coagulated with the hemostatic forceps in the soft coagulation mode and then resected with the forceps in the endocut mode. Moreover, the coagulated submucosa surrounding the vessel is also resected with the forceps. Removing the coagulated vessel and the surrounding submucosa ensures that the subsequent submucosal dissection is safer and easier than otherwise. When massive bleeding that cannot be stopped by the knife occurs during ESD, special hemostatic forceps are used in the soft coagulation mode as described above. Endoscopic clipping is performed when bleeding cannot be controlled with the special forceps. 

**Figure 5:**
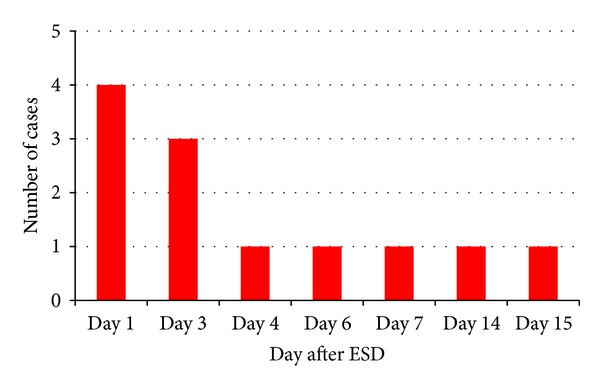
Periods of occurrence of postoperative hemorrhage.

**Figure 6:**
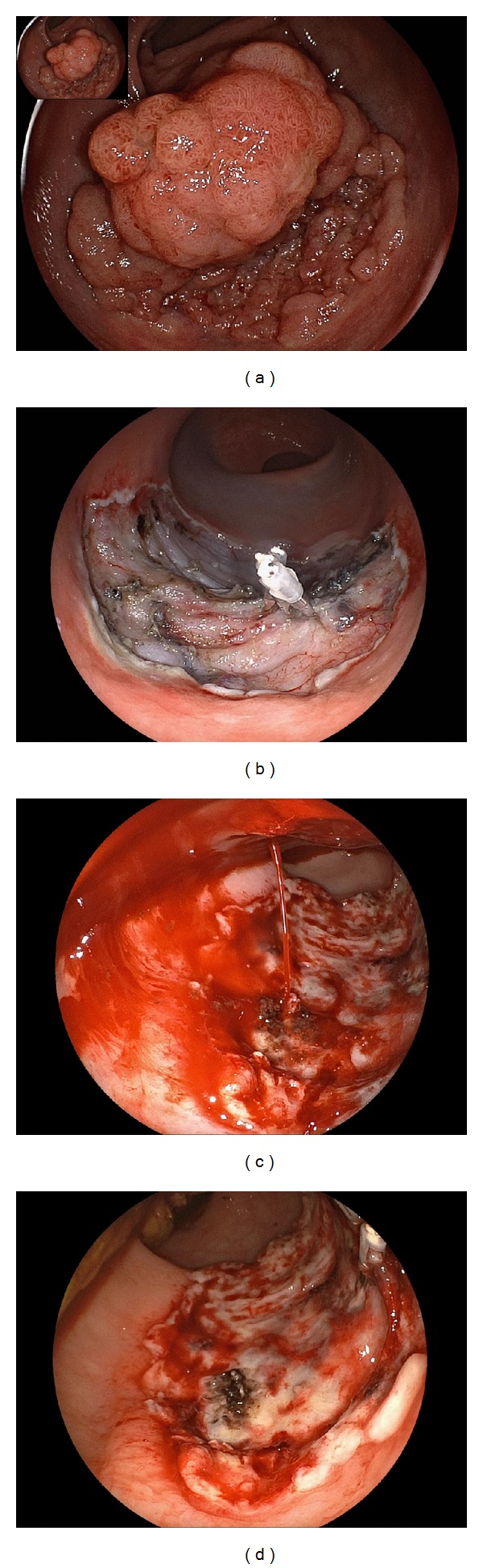
(a) Rectal tumor, 0-IIa, 50 mm in diameter. (b) The ulceration after endoscopic submucosal dissection (ESD). (c) Pulsing hemorrhage occurred 4 days after ESD. (d) The vessel was coagulated by hemostatic forceps.

The rate of postoperative hemorrhage in ESD is reported to be 0–12.0% [1–9, 12]. This rate is comparable to that reported for EMR [19–21]. Our analysis showed the period of occurrence of postoperative hemorrhage (Figure 5). Within 3 days after ESD, postoperative hemorrhage occurred in 7 of 12 cases (58.3%). An anticoagulant was prescribed for 3 cases in which postoperative hemorrhage occurred at >7 days after ESD. The majority of cases of postoperative hemorrhage are treated only by endoscopic clipping or endoscopic coagulation, and by withholding oral intake without emergency surgery or blood transfusion (Figure 6) [22]. In our study, 6 cases were treated by endoscopic clipping and 6 cases were treated by endoscopic coagulation with hemostatic forceps.

## 5. Other Complications

**Figure 7:**
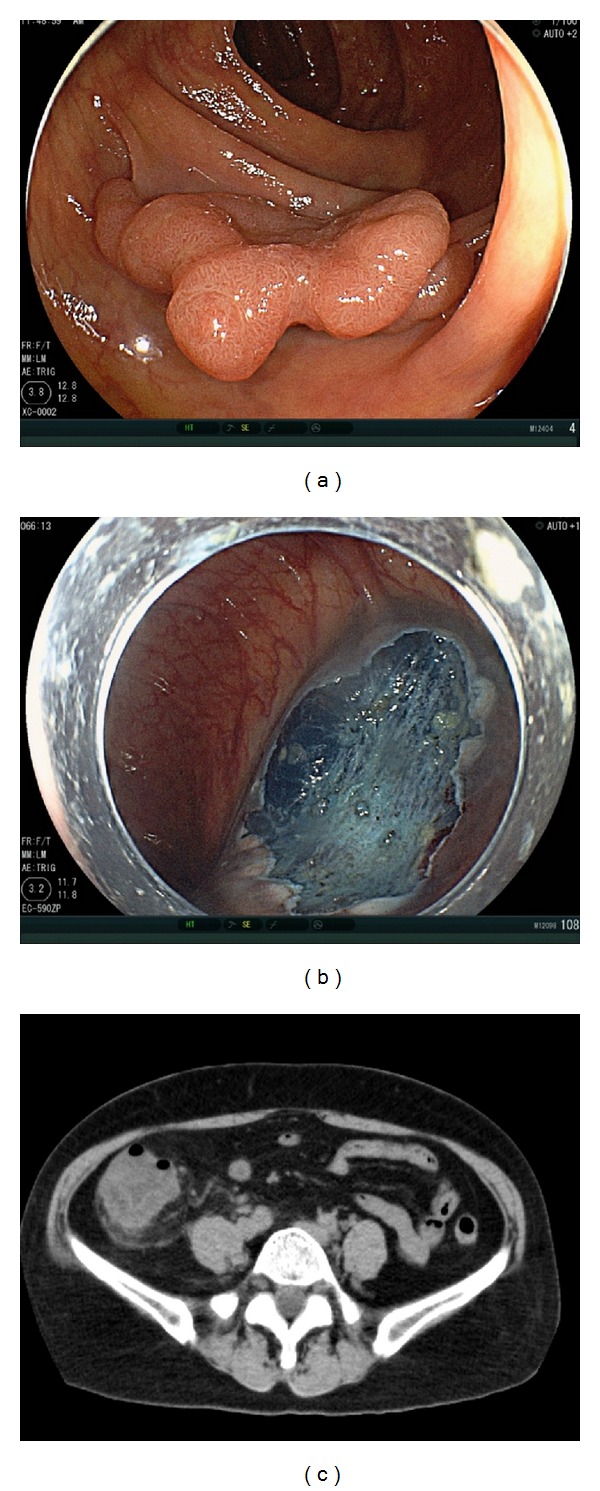
(a) Colonic tumor, 0-IIa, 40 mm in diameter, located in the ascending colon. (b) The ulceration after endoscopic submucosal dissection (ESD). ESD was performed without perforation and coagulation of the muscle layer. (c) Abdominal computed tomography 2 days after ESD revealed wall thickness on the whole ascending colon.

Inflammation has been reported to occur to a certain degree in some cases. In our previous report, the mean concentration of C-reactive protein at 2 days after ESD was 5.82 ± 12.10 mg/L in cases with perforation and 1.27 ± 2.00 mg/L in cases without perforation [7]. At our institution, severe local peritonitis without perforation was seen in 4 of 518 cases (0.7%). The concentration of C-reactive protein was >15 mg/L in all 3 cases. Computed tomography showed whole wall thickness around the ESD locus (Figure 7) [22]. This was considered to be possibly due to coagulation of the muscle layer. 

Severe restlessness of the patient owing to abdominal fullness and pain has rendered submucosal dissection impossible in some cases. Conscious sedation to prevent restlessness is effective for some patients. Carbon dioxide insufflation has been reported to be effective for the prevention of abdominal fullness [23]. At our institution, conscious sedation is performed with midazolam (Dormicum; Astellas Pharma Inc., Tokyo, Japan) and pentazocine (Pentajin; Daiichi Sankyo Co., Tokyo, Japan) with monitoring with an automatic blood pressure monitor. In our previous ESD study that included 105 cases, there were 22 patients for whom the operation time exceeded 2.5 h, and 15 of these 22 patients (68.1%) experienced restlessness despite conscious sedation [15]. In contrast, among the 83 patients with an operation time of <2.5 h, restlessness occurred in only 10 (12.0%). Thus, restlessness due to abdominal fullness and pain occurs frequently in patients with an operation time exceeding 2.5 h. 

## 6. Training for ESD

**Figure 8:**
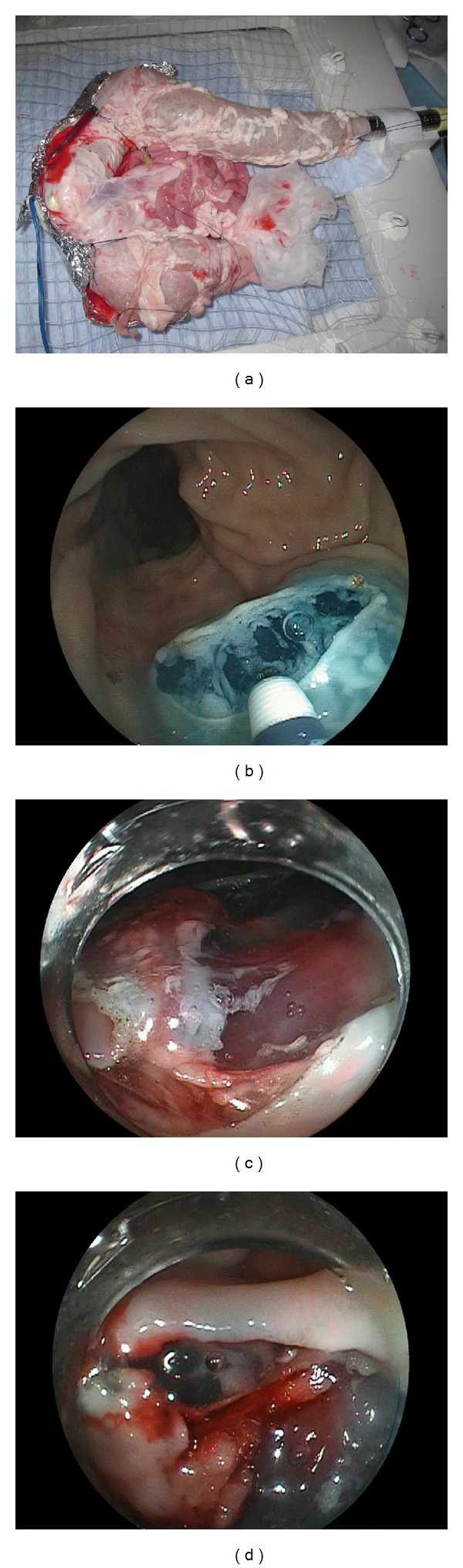
(a) *Ex vivo* blood flow animal model (Johnson & Johnson, Tokyo, Japan) for training for endoscopic hemostasis in colorectal endoscopic submucosal dissection. (b) Submucosal dissection was performed. (c) In the *ex vivo* blood flow model (bovine cecum), the submucosal vessels were visible. (d) Perioperative hemorrhage was stopped endoscopic coagulation with a knife.

Colorectal ESD is difficult for less-experienced endoscopists. In general, endoscopists should first acquire extensive experience with gastric ESD before performing colorectal ESD. However, a different training system for colorectal ESD is required when there are few patients with early gastric cancer, as in Western countries. In such situations, training could consist of visiting ESD experts at other institutions and observing them at work. Another expected component of ESD training is extensive practice on animal models [24–26]. Both *in vivo* and *ex vivo* (using harvested organs) animal models have been used. Porcine and canine *in vivo* models have been reported to be useful systems for ESD training [24–26]. However, *in vivo* animal models are expensive and difficult to prepare. Parra-Blanco et al. [25] demonstrated the usefulness of a porcine colon *ex vivo* animal model for training in colorectal ESD. Additionally, we have reported the evaluation of various *ex vivo* animal models, and an *ex vivo* animal model with simulated blood flow with the help of Johnson & Johnson [27]. In the study, the porcine cecum, rectum, and stomach, and the bovine cecum and rectum were evaluated in view of mucosal injection, submucosal elevation, and status of the muscle layer. Each *ex vivo* animal model has characteristic features, making it possible to choose a suitable model according to the skill level of the endoscopist. On the basis of its characteristics, we recommend the bovine rectum for training beginners in colorectal ESD because mucosal injection is easy, submucosal elevation is high, and the muscle layer is tight in this model. The blood flow model can be made using the bovine cecum (Figure 8). The vessel around the cecum is detached and red ink is injected. This model can allow the endoscopist to gain the whole ESD experience, including perioperative hemorrhage. We use this model not only for ESD training of less experienced endoscopists but also for training for the use of new devices. 

A unique step-by-step ESD training system has been implemented at some Japanese institutions, including ours [27]. This system starts with observing and assisting in ESD procedures performed by experts. Next, animal model training is performed to the extent possible. Finally, clinical practice is performed under the supervision of instructors. Generally, clinical practice training proceeds according to the difficulty of the procedure, beginning with gastric ESD, then rectal ESD, and finally colonic ESD [27]. In Western countries where the number of patients with early gastric cancer is few, Uraoka et al. [28] proposed the special step-by-step training system, including training with animal models, in accumulating colorectal ESD experience. In their report, they suggested that ESD training with animal models should be supervised by Japanese and Western experts. We also strongly recommend this system because we believe beginners could not achieve an accurate technique without the supervision of experts. In clinical ESD training, it is recommended to start with laterally spreading, granular-type tumors 2-3 cm in diameter located in the rectum. Such tumors have a low risk of submucosal invasion. Additionally, these tumors are easy to dissect because they allow flexible movement of the endoscope. 

Concerning animal model training, we believe that the experience from training on an animal model will also improve the endoscopist's performance in clinical colorectal ESD; however, this is difficult to prove theoretically. Repeated training on animal models has recently been proven to decrease procedure time [24–26, 29, 30]. One interesting study from Italy about stepwise training for ESD reported that a minimal intensive training based on animal models and expert exchanges enabled endoscopists expert in other therapeutic procedures to perform ESD [31]. The rectal ESD learning curve demonstrated the feasibility and safety of the procedure with a competence threshold set at 20 procedures. However, the colonic ESD learning curve showed extremely low *en bloc* resection rates and a high perforation risk in the early phase, although competence was achieved after 20 procedures. In Japan, Hotta et al. [32] showed that approximately 40 procedures were sufficient to acquire skill in avoiding perforations, and the perforation rate in the first 40 cases was about 12.5%. On the other hand, Sakamoto et al. [33] demonstrated that 30 colorectal ESD procedures are required to achieve self-sufficiency. In view of these Japanese reports, the Italian report about stepwise training showed the effectiveness of training on animal models for clinical ESD. Additionally, we have proved the usefulness of the expertise in endoscopic closure gained from training on *ex vivo* animal models [27]. The completion rate and procedure time for endoscopic clipping were improved only in nonexpert endoscopists during 10 practice procedures on an *ex vivo* animal model. 

## 7. Conclusions

 ESD is a feasible technique because it is an efficient treatment for large colorectal tumors and is less invasive than surgical operation. Endoscopists have to know the complications of colorectal ESD in order to prevent them. Additionally, appropriate training, including animal model training, for colorectal ESD should be acquired before working on clinical cases.

## Figures and Tables

**Table 1:** Clinicopathological factors of 530 colorectal ESDs in our institution.

Clinicopathological factors	*N* = 530
Tumor size (range) (mm)	31.2 (10–130)
Location	Colon: 364 (68.7%) Rectum: 166 (31.3%)
Morphology	Protruding: 102 (19.2%) Superficial: 428 (80.8%)
Rate of *en bloc* resection	91.1%, 483/530
Procedure time (min) (*N* = 517)	93.4 (10–420)
Histopathological diagnosis (*N* = 517)	Ad: 236 (45.6%) M: 218 (42.2%) SM: 63 (12.2%)
Stop of ESD	2.5%, 13/530
Postoperative hemorrhage	2.3%, 12/530
Perforation	4.1%, 22/530
Local severe peritonitis without perforation	0.7%, 4/530

Ad: adenoma, M: intramucosal cancer, SM: submucosally invaded cancer.
